# Investigation of improving the pre-training and fine-tuning of BERT model for biomedical relation extraction

**DOI:** 10.1186/s12859-022-04642-w

**Published:** 2022-04-04

**Authors:** Peng Su, K. Vijay-Shanker

**Affiliations:** Department of Computer and Information Science, Biomedical Text Mining Lab, University of Delaware, Newark, USA

**Keywords:** Deep learning, Transformer, BERT, Text mining, Biomedical relation extraction

## Abstract

**Background:**

Recently, automatically extracting biomedical relations has been a significant subject in biomedical research due to the rapid growth of biomedical literature. Since the adaptation to the biomedical domain, the transformer-based BERT models have produced leading results on many biomedical natural language processing tasks. In this work, we will explore the approaches to improve the BERT model for relation extraction tasks in both the pre-training and fine-tuning stages of its applications. In the pre-training stage, we add another level of BERT adaptation on sub-domain data to bridge the gap between domain knowledge and task-specific knowledge. Also, we propose methods to incorporate the ignored knowledge in the last layer of BERT to improve its fine-tuning.

**Results:**

The experiment results demonstrate that our approaches for pre-training and fine-tuning can improve the BERT model performance. After combining the two proposed techniques, our approach outperforms the original BERT models with averaged F1 score improvement of 2.1% on relation extraction tasks. Moreover, our approach achieves state-of-the-art performance on three relation extraction benchmark datasets.

**Conclusions:**

The extra pre-training step on sub-domain data can help the BERT model generalization on specific tasks, and our proposed fine-tuning mechanism could utilize the knowledge in the last layer of BERT to boost the model performance. Furthermore, the combination of these two approaches further improves the performance of BERT model on the relation extraction tasks.

## Background

In recent years, biomedical text mining has become an urgent problem since the manual curation lags far behind the explosive growth of biomedical literature. In biomedical text mining, relation extraction (RE) is an important task that aims to identify the relations between biomedical entities mentioned in the text. Substantial techniques have been developing to extract biomedical relations such as protein-protein interactions (PPI [1]), drug-drug interactions (DDI [2]), chemical protein interactions (ChemProt [3]). After the extraction of different relations, we can convert the unstructured literature to structured information, which is a crucial step towards natural language understanding applications like automated reasoning [4], machine translation [5], question answering [6], etc.

Extraction of various relations in biomedical domain has attracted tremendous attentions and many different methods have been proposed [7–11]. Recently, language model methods dominate the relation extraction field with their superior performance [12–15]. Applying language models on relation extraction problem includes two steps: the pre-training and the fine-tuning. In the pre-training step, a vast amount of unlabeled data can be utilized to learn a language representation. The fine-tuning step is to learn the knowledge in task-specific (labeled) datasets through supervised learning. Among all the language models, BERT [14]—Bidirectional Encoder Representations from Transformers [16], attracts lots of attentions from researchers in different fields.

BERT is designed to learn a universal and context-dependent language representation using the transformer encoder [16]. Originally, BERT was proposed to learn the representation for general domain. To make the model generalize better in biomedical domain, several BERT adaptations for the biomedical domain have been proposed such as BioBERT [17], BlueBERT [18], SciBERT [19], and PubMedBERT [20]. BioBERT [17] further pre-trains the BERT model on PubMed abstracts and PMC full-length articles; BlueBERT [18] is further pre-trained on PubMed abstracts and MIMIC-III clinical notes [21]; A collection of 1.14M articles from Semantic Scholar [22] are used to pre-train SciBERT model [19]. While the first three biomedical BERT models are pre-trained on the basis of the original BERT, PubMedBERT [20] is pre-trained with whole-word masking from scratch using PubMed abstracts.

In this work, we propose and evaluate two approaches to improve the BERT-style model on relation extraction tasks. We first consider the approach for the pre-training phase and later discuss the other that is related to the fine-tuning phase. Our goal to improve the pre-training of BERT model is motivated by the results of the experiments we conducted to investigate differing performances of several BERT-based models for the biomedical domain. Different models such as BioBERT [17], SciBERT [19] and BlueBERT [18] are developed for the biomedical domain. One of the primary differences between them is the corpora used in the pre-training for domain adaptation. Those pre-trained BERT models are suppose to have similar performance on similar applications since they were pre-trained on similar biomedical data. However, our experiments reveal that those models have significantly different performance on the same set of relation extraction tasks (Table 2). Therefore, we hypothesize that the text used in the pre-training for domain adaptation can have a significant impact on the downstream applications.

In order to leverage the pre-training for a specific task, we introduce another level of adaptation to adjust the domain adaptation to specific sub-domains in this work. We call this part sub-domain adaptation. To fulfill this task, we add one more pre-training step on sub-domain data. For example, for a relation extraction task like the extraction of drug-drug interactions (DDI), we investigate whether adding more drug-specific text can help over general biomedical domain knowledge. After the sub-domain adaptation, we expect that the pre-trained BERT model will generalize better on the specific tasks. There are several studies using the labeled training data in the pre-training of language model for better generalization [23, 24]. However those methods are not feasible for our tasks since we also need the context of the training sentences (for Next Sentence Prediction in pre-training of BERT). Thus, we propose a new approach to acquire more general pre-training data for our tasks.

Our motivation to improve the fine-tuning of BERT is from the following observations. All previous work utilizing BERT-based models for relation extraction tasks employ a standard way of fine-tuning using the classification token (CLS) alone among the last layer. Thereby all information contained in other final layer nodes is completely ignored during the fine-tuning process. However, the ignored knowledge in the last layer of BERT model is utilized for other tasks like sequence tagging. From this point of view, the BERT model is fine-tuned with one less layer for classification tasks. This drives us to design a new mechanism of using all the information in the last layer for the classification tasks. There are several studies that apply additional layers on the outputs of BERT like Sentence-BERT [25], proteinBERT [26]. Our work differs from the previous work in the following aspects: (1) we demonstrate the usefulness of the knowledge in the last layer of BERT utilizing the probing technique; (2) we investigate three different methods (LSTM, biLSTM, and attention mechanism) to summarize the information in the last layer; (3) we provide some evidence to explain why the attention mechanism is performing better than the other two methods.

In addition, our investigation of improving fine-tuning mechanism is partially driven by the insights in [27], in which the authors find that the BERT model learns the representation similar to traditional natural language processing (NLP) pipelines. Based on the findings in [27], the basic syntactic aspects of the text appear to be learned in the lower layers, while high-level semantic information appears in the higher layers of BERT. Since relation extraction tasks are concerned with the semantic relations between entities, we were curious about whether the upper (including top) layers contain important information about relation extraction tasks. During this investigation, we employ the edge probing technique [28] to measure how much relevant information (about relation extraction tasks) the last layer contains. The results illustrate that the last layer of BERT model contains useful information, but it is unused in the original fine-tuning method of only using classification (CLS) token. To incorporate the unused information from the last layer into fine-tuning, we explore two different methods: recurrent neural network (RNN) and attention mechanism [29]. The summarized knowledge will be concatenated with the classification token as the model output in an improved fine-tuning mechanism. We call it fine-tuning with information summarization of the last layer (SLL fine-tuning).

To demonstrate the effectiveness of our proposed approaches in the pre-training and fine-tuning process, we evaluate them on three extensively studied relation extraction tasks in biomedical field: protein-protein interactions (PPI [1]), drug-drug interactions (DDI [2]), and chemical-protein interactions (ChemProt [3]). The experiment results illustrate that both sub-domain adaptation and the proposed fine-tuning mechanism can boost the model performance on all the tasks. In addition, the combination of these two approaches outperform previous methods on the three benchmark datasets.

In summary, the contributions of this work are:

- Demonstrating that further adaptation on sub-domain data can improve the pre-training of BERT model for specific tasks;
- Utilizing the edge probing technique to explore the ignored knowledge in the last layer of BERT model;
- The SLL fine-tuning mechanism is proposed to utilize all the available knowledge in the last layer to boost the BERT model performance;
- State-of-the-art performance is achieved on three relation extraction benchmark datasets.

## Results and discussion

In this work, we experiment with two types of BERT model adapted for the biomedical domain: (1) the BERT model adapted from general domain using biomedical text (e.g., BioBERT [17], BlueBERT [18], SciBERT [19]); (2) the BERT model adapted from scratch using biomedical text (e.g., PubMedBERT [20]). Our study starts with the comparison of BERT models from the first type. Then we experiment with both types of BERT models on the proposed approaches. We choose BioBERT from the first type of BERT models as it generally outperforms SciBERT and BlueBERT on our tasks (Table 2). For the second type of BERT model, we use PubMedBERT in our experiments.

We verify the effectiveness of the proposed approaches on three benchmark datasets and we show the statistics of these datasets in Table 1. We use the AIMed corpus [30] for the PPI task. For the ChemProt and DDI tasks, we use the datasets in [2, 3], respectively. We employ the the same spilt of training, development, and test sets with the PubMedBERT model [20] during the model evaluation. For the AIMed corpus, standard sets of training and test are not available, so we apply 10-fold cross-validation during evaluation. We employ precision (P), recall (R) and F1-score (F) to evaluate the model on the PPI task since it is a binary classification problem. However, the models for ChemProt and DDI tasks will be evaluated with micro precision (P), recall (R) and F1 score (F) on the non-negative classes since they are multi-class classification problems.

**Table 1:** Datasets statistics for PPI, DDI, and ChemProt

Dataset	Instance #	Train	Dev	Test
PPI(AIMed)	5,834	–	–	–
DDI	33,508	22,233	5559	5716
ChemProt	45,048	18,035	11,268	15,745

For the AIMed dataset of PPI, there are only two labels: Positive and Negative. The ChemProt corpus is labeled with five positive classes (CPR:3, CPR:4, CPR:5, CPR:6, CPR:9) and the negative class. Similarly, the DDI dataset contains four positive labels (ADVICE, EFFECT, INT, MECHANISM) and one negative label

In this section, we first discuss the effect of pre-training corpora on the BERT models. Next, the experiment results on sub-domain adaptation of BERT models are demonstrated. Then, we present the results of exploring the learned knowledge in the last layer and the SLL fine-tuning. Also, the model performance of combining the proposed approaches for the pre-training and fine-tuning is provided for the three tasks. Finally, we perform an analysis on the learned attention weights from our SLL fine-tuning.

### Impact of corpus on domain adaptation of different BERT models

We begin with a study of the impact of the corpora used to adapt BERT-based models to the biomedical domain. Specifically, we experiment with the three well-known models: BioBERT [17], BlueBERT [18] and SciBERT [19]. Considering these three models have the same architecture, the primary difference between them is the corpus used to adapt to the biomedical domain during pre-training. These three models are supposed to have similar performance, but the results are contrary to our expectation as they are summarized in Table 2. BlueBERT shows performance drop-off compared to the other two on our three datasets, especially on the PPI set. Thus, the text used in pre-training for domain adaptation appears to have a surprisingly significant influence on the performance of BERT models on downstream tasks. A noticeable difference with BlueBERT, when compared to the other two models, is the inclusion of clinical notes text in the domain adaptation process, which differs considerably from the text used for pre-training other two models. Since all three evaluation sets are irrelevant to the clinical domain, we conduct an experiment to see if the removal of this extraneous material from pre-training adaptation would impact the results. The results, shown in Table 3, suggest an improvement in BlueBERT’s performance (BlueBERT almost achieves the same results as the other two). This observation leads us to conjecture that task-related data in the pre-training might yield better generalization of BERT models on downstream tasks.

**Table 2:** Performance (F1 score) of BERT models on the ChemProt, DDI, and PPI datasets

Model	PPI	DDI	ChemProt
BioBERT	81.0	79.0	75.3
SciBERT	78.8	78.7	74.4
BlueBERT	71.9	76.8	71.2

**Table 3:** Performance of BlueBERT model on the PPI, ChemProt, and DDI tasks before and after removing MIMIC-III from the domain adaptation data

Model	PPI			DDI			ChemProt		
	P	R	F	P	R	F	P	R	F
BlueBERT	69.3	75.0	71.9	76.2	77.4	76.8	70.9	71.5	71.2
BlueBERT (-M)	**76.6**	**83.1**	**79.6**	**80.0**	**78.5**	**79.2**	**74.7**	**75.8**	**75.2**

Bold values indicate better results

P: Precision; R: Recall; F: F1 Score; -M: Subtract the MIMIC-III clinical notes

### Sub-domain adaptation

While all BERT models try to use a large corpus of text from the biomedical domain to obtain the domain-adapted versions, our results indicate that the differences between the domain adaptation text might have a noticeable impact on individual tasks, as each task requires its own specific knowledge. Therefore, we wish to further consider the pre-training data for the pre-training phase.

Given our investigation involving several relation extraction tasks, we consider the approach of utilizing task-related data to further adjust the adaptation for the domain underlying the tasks. Specifically, we extract data (unlabeled text) for three sub-domains from PubMed for our tasks: protein/gene (P/G) domain, drug (D) domain and chemical+protein (CP) domain. These three sets of abstracts are used for sub-domain adaptation setup to produce three differently adapted BERT models: BERT(+P/G), BERT(+D) and BERT(+CP), respectively.

Table 4 presents the results of our experiments on sub-domain adaptation. The results are reported for both BioBERT and PubMedBERT, which have already been adapted to the biomedical domain. Their performances are shown in the first row and the fifth row, and serve as baselines to compare with those models that have additional pre-training for sub-domain adaptation. The other results in Table 4 show that the sub-domain adaptation can boost the BERT model performance on related tasks. Note that not only are similar results obtained for BioBERT and PubMedBERT, but also for each task, the addition of sub-domain adaptation phase improves the performance. Furthermore, the maximum improvement is obtained when the most relevant sub-domain text is used in the sub-domain adaptation phase. Specifically, the best results are obtained for the PPI task by using “protein/gene” related text. Also, the “drug” text leads to the highest performance for the DDI task. Both BioBERT and PubMedBERT obtain maximum benefits in the use of “chemical+protein” text for sub-domain adaptation on the ChemProt task. On the other hand, adding drug related text does not help for the PPI task. Also, the addition of “protein/gene” text actually hurts the performance of both models when used for the DDI task.

**Table 4:** BERT performance after pre-training with sub-domain data

Model	PPI			DDI			ChemProt		
	P	R	F	P	R	F	P	R	F
BioBERT	79.0	83.3	81.0	79.9	78.1	79.0	74.3	**76.3**	75.3
BioBERT (+P/G)	${\mathbf{82.5 }}$	${\mathbf{83.7 }}$	${\mathbf{83.0 }}$	76.1	77.6	76.9	76.5	74.2	75.3
BioBERT (+D)	81.5	80.9	81.2	${\mathbf{81.9 }}$	${\text {78.4}}$	${\mathbf{80.1 }}$	**76.7**	74.4	75.6
BioBERT (+CP)	81.3	83.7	82.4	78.7	$\mathbf{79}.0$	78.8	${\text {76.6}}$	${\text {76.1}}$	${\mathbf{76.4 }}$
PubMedBERT	80.1	84.3	82.1	82.6	81.9	82.3	78.8	75.9	77.3
PubMedBERT (+P/G)	${\mathbf{81.2 }}$	${\mathbf{85.5 }}$	${\mathbf{83.3 }}$	83.7	80.5	82.0	**80.5**	75.5	77.9
PubMedBERT (+D)	79.1	85.3	82.0	${\mathbf{84.1 }}$	${\text {81.7}}$	${\mathbf{82.9 }}$	80.4	74.6	77.4
PubMedBERT (+CP)	79.6	84.7	82.0	81.1	**82.7**	81.9	${\text {79.4}}$	${\mathbf{77.5 }}$	${\mathbf{78.4 }}$

Bold values indicate better results

P: Precision; R: Recall; F: F1 Score; +P/G: add Protein/Gene-related PubMed abstracts as sub-domain data; +D: add Drug-related PubMed abstracts as sub-domain data; +CP: add protein-related and chemical-related PubMed abstracts as sub-domain data

All the experiment results indicate that the pre-trained BERT models generalize differently on different tasks, and it is beneficial to add another level of adaptation on task-specific data. In this work, we have demonstrated that using entity-related data in the sub-domain adaptation helps the model generalization on relation extraction tasks.

### Learned knowledge in the last layer of BERT

**Fig. 1:**
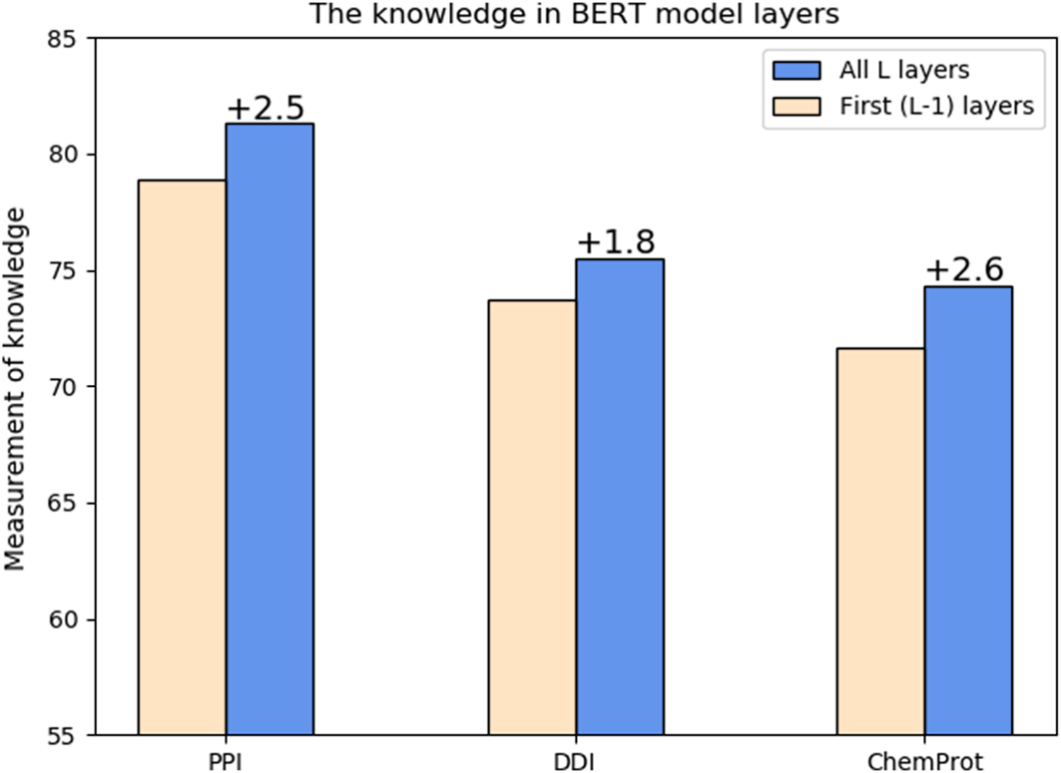
Learned knowledge of training data in the layers of BioBERT. L is the total layers of BERT model. “Measurement of Knowledge” ($\Delta _\tau ^L$) is defined in the Methods section

In this subsection, we focus on improving the fine-tuning process of BERT. Using the technique of probing classifier, we show that the last layer of BERT model contains useful information that can be exploited to improve performance on downstream tasks (Fig. 1). Specifically, we compare the performance of probing classifiers using all L layers and first (L-1)-th layers to measure the knowledge captured in the last layer (not present in the previous layers). We can see that the probing classifier using the information in all L layers performs better on all three tasks, which means many instances are predicted correctly by adding the knowledge in the last (L-th) layer, but not using the knowledge in the first (L-1)-th layers. Thus it is beneficial to utilize those information during fine-tuning of BERT. In addition, the outputs from the last layer are automatically computed during training and inference, we can just utilize them without extra cost.

### SLL fine-tuning: utilizing the summarized information in the last layer

Having shown that the last layer contains some useful information, which is not exploited by current transformer-based models for RE, we consider two different methods to utilize it and incorporate the summarized information in a refined fine-tuning process. In Table 5, we provide the model performance using different methods: RNN models (both LSTM and biLSTM) and attention mechanism. Clearly, the method of applying attention mechanism on the outputs of last layer (BERT_SLL_Att) obtains the best performance on the three tasks. Specifically, the attention-based method on BioBERT model achieves F1 score improvement of 1.5%, 1.5%, and 1.0% on PPI, DDI, and ChemProt, respectively. Similarly, we also observe F1 improvements with the PubMedBERT model for the three tasks.

**Table 5:** Performance of BERT models on PPI, DDI, and ChemProt.

Model	PPI			DDI			ChemProt		
	P	R	F	P	R	F	P	R	F
BioBERT	79.0	83.3	81.0	79.9	78.1	79.0	74.3	**76.3**	75.3
BioBERT_SLL_LSTM	80.2	84.0	82.0	80.5	78.5	79.5	77.6	74.4	76.0
BioBERT_SLL_biLSTM	80.2	82.7	81.4	80.8	78.5	79.6	**77.9**	73.9	75.9
BioBERT_SLL_Att	**80.7**	**84.4**	**82.5**	**81.6**	**79.4**	**80.5**	77.5	75.1	**76.3**
PubMedBERT	80.1	84.3	82.1	82.6	81.9	82.3	78.8	75.9	77.3
PubMedBERT_SLL_LSTM	79.8	**85.6**	82.6	82.6	**82.8**	82.7	**78.9**	77.0	**77.9**
PubMedBERT_SLL_biLSTM	80.5	82.6	81.7	82.6	81.4	82.0	78.5	76.5	77.5
PubMedBERT_SLL_Att	**81.3**	85.0	**83.1**	**84.3**	82.7	**83.5**	78.3	**77.6**	**77.9**

Bold values indicate better results

P: Precision; R: Recall; F: F1 Score; BioBERT/PubMedBERT_SLL_LSTM: model of summarizing the outputs of the last layer using LSTM; BioBERT/PubMedBERT_SLL_biLSTM: model of summarizing the outputs of the last layer using biLSTM; BioBERT/PubMedBERT_SLL_Att: model of summarizing the outputs of the last layer using attention mechanism

Even though the LSTM and biLSTM methods show less improvement compared to the use of attention mechanism, they improve the model performance in most of the cases. We also observe that LSTM and biLSTM have very similar performance, with LSTM being slightly better in general. Since the training of BERT considers the context of words from both directions, we hypothesize that the backward encoding in biLSTM will not provide extra information for the tasks(which partially explains the experiment results of LSTM and biLSTM). Therefore, we will only utilize LSTM when considering RNN model for the experiments in the following subsections.

### Combining sub-domain adaptation and SLL fine-tuning mechanism

The two proposed techniques can be combined as they are for two independent stages of BERT model. As shown in Table 6, combining the sub-domain adaptation and the proposed fine-tuning mechanism can further boost the model performance on all the three tasks. The first two rows for BioBERT and PubMedBERT are just repetitions of the model performance from Table 5 and serve as the baselines for the results of using combined techniques. In comparison with the original BioBERT model, the model with combined techniques can improve the F1 score with 2.8%, 2.9%, and 1.7% on the PPI, DDI, and ChemProt tasks, respectively. Similarly, we can also boost the PubMedBERT model performance with 1.9%, 1.7%, and 1.4% F1 score improvement on the three tasks, respectively. As far as we know, PubMedBERT is the state-of-the-art model for these three tasks. After further improving its performance, we now achieve the state-of-the-art performance on all three benchmark datasets.

**Table 6:** BERT performance after combining sub-domain adaptation and the refined fine-tuning mechanism

Model	PPI			DDI			ChemProt		
	P	R	F	P	R	F	P	R	F
BioBERT	9.0	83.3	81.0	9.9	8.1	9.0	4.3	6.3	5.3
BioBERT_SLL_Att	80.7	84.4	82.5	81.3	80.1	80.7	76.5	**77.1**	76.8
BioBERT_SLL_Att (+P/G)	${\mathbf{83.1 }}$	${\mathbf{84.7 }}$	${\mathbf{83.8 }}$	80.4	79.7	80.0	78.4	75.1	76.7
BioBERT_SLL_Att (+D)	81.5	84.5	82.9	${\mathbf{82.6 }}$	${\mathbf{81.2 }}$	${\mathbf{81.9 }}$	76.8	74.7	75.7
BioBERT_SLL_Att (+CP)	82.5	84.2	83.3	81.7	77.0	79.3	${\mathbf{78.9 }}$	${\hbox {75.2}}$	${\mathbf{77.0 }}$
PubMedBERT	80.1	84.3	82.1	82.6	81.9	82.3	78.8	75.9	77.3
PubMedBERT_SLL_Att	81.3	85.0	83.1	84.3	82.7	83.5	78.3	77.6	77.9
PubMedBERT_SLL_Att (+P/G)	${\hbox {81.1}}$	${\mathbf{87.1 }}$	${\mathbf{84.0 }}$	83.6	80.6	82.1	**79.8**	77.0	78.4
PubMedBERT_SLL_Att (+D)	**81.4**	84.5	82.9	${\hbox {84.9}}$	${\mathbf{83.2 }}$	${\mathbf{84.0 }}$	79.5	75.9	77.7
PubMedBERT_SLL_Att (+CP)	**81.4**	85.7	83.4	**85.0**	81.4	83.2	${\hbox {79.7}}$	${\mathbf{77.7 }}$	${\mathbf{78.7 }}$

Bold values indicate better results

P: Precision; R: Recall; F: F1 Score; BioBERT/PubMedBERT: original BERT model; BioBERT/PubMedBERT_SLL_Att: model of summarizing the outputs of the last layer using attention mechanism. +P/G: add Protein/Gene-related PubMed abstracts as sub-domain data; +D: add Drug-related PubMed abstracts as sub-domain data; +CP: add protein-related and chemical-related PubMed abstracts as sub-domain data

**Table 7:** Model performance without using the [CLS] token in the last layer

Model	PPI			DDI			ChemProt		
	P	R	F	P	R	F	P	R	F
BioBERT	79.0	83.3	81.0	79.9	78.1	79.0	74.3	76.3	75.3
BioBERT_SLL_Att	80.7	**84.4**	82.5	**81.3**	80.1	**80.7**	**76.5**	**77.1**	**76.8**
BioBERT_SLL_Att*	**82.3**	83.5	**82.8**	79.7	77.6	78.6	76.4	74.5	75.4
PubMedBERT	80.1	84.3	82.1	82.6	81.9	82.3	**78.8**	75.9	77.3
PubMedBERT_SLL_Att	**81.3**	85.0	**83.1**	**84.3**	**82.7**	**83.5**	78.3	77.6	**77.9**
PubMedBERT_SLL_Att*	80.0	**85.2**	82.4	82.5	80.9	81.7	75.7	**77.7**	76.7

Bold values indicate better results

P: Precision; R: Recall; F: F1 Score; BERT_SLL_Att*: models of fine-tuning with only the summarized information from attention mechanism (without [CLS] token)

### More analysis: roles of classification ([CLS]) token in fine-tuning

We have shown that the proposed fine-tuning method outperforms original fine-tuning mechanism in Table 5. Both methods include the use of [CLS] token in the last layer, so it will be helpful to understand the role of [CLS] token. Here we experiment with the model of fine-tuning without [CLS] token to investigate the contribution of [CLS] token. In particular, we drop the [CLS] output and only utilize the summarized information from attention mechanism as the output of BERT model in both training and inference.

Here we experiment with both BioBERT and PubMedBERT models. In Table 7, the third and sixth row show the model performance of employing only the summarized outputs in the last layer (i.e., without [CLS] output). The four rows before (row 1, 2, 4, 5) are only repetitions of the model performance from Table 5. We observe that fine-tuning without using [CLS] token hurts the model performance on most cases and only BioBERT_SLL_Att* model performs slightly better on the PPI task. These results show that the [CLS] token in the last layer contains important information about our classification tasks.

In addition, the authors in [31] explore the contribution of [CLS] tokens from the intermediate layers of BERT, we also experiment with this method and observe worse model performance after incorporating the [CLS] outputs from the intermediate layers in all tasks. Thus, we will not present those results here. Our experiment results imply that it is better to use information from the last layer since the knowledge in the intermediate layers will be transferred to the latter layers (through the residual connection) during training and inference.

### Analysis of attention weights in the SLL fine-tuning

**Fig. 2:**
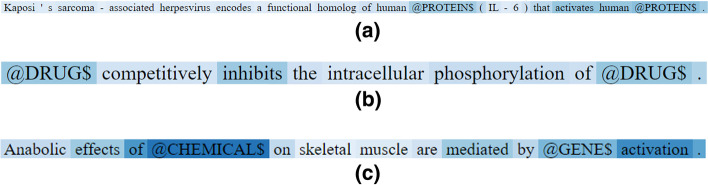
The visualization of attention weights in the last layer. **a** PPI example; **b** DDI example; **c** ChemProt example

In the previous subsection, we illustrate that utilizing additional attention mechanism yields better performance. We conduct some preliminary experiments to understand whether the additional mechanism focuses on specific parts of the input text for our tasks. We first visualize the weight distribution (from the attention mechanism) on the words (tokens) of the sentences and inspect which words (tokens) the model is focusing on. In Fig. 2, we have chosen three examples from the three tasks (one example for each task) that were predicted incorrectly by the original BioBERT but are correctly classified by the BioBERT model with SLL fine-tuning. In Fig. 2, the darkness of the color represents the attention weight, which means the words with darker color have larger attention weights.

Figure 2 demonstrates that the attention mechanism is assigning relatively large weights on “informative” words (which express the relationship of the entities), which are usually called “trigger words” for the relations in the NLP field. It appears that the attention mechanism is learning to focus on the “trigger words” in our tasks when making its predictions. For instance, in the example of PPI relation (Fig. 2a), the attention mechanism is assigning larger weights on the entity token (i.e., @PROTEIN$) and the trigger word (i.e., “activates”). The attention mechanism, likewise, assigns larger weights on the trigger words (“inhibit” and “mediate”) in the examples of DDI and ChemProt, respectively. Considering the original BioBERT model misclassified these sentences, those examples also show that the attention mechanism is able to learn the knowledge (especially about trigger words) of relation expression.

Previously, we demonstrate that the attention mechanism assigns large weights to relation expression words (trigger word and entity token) through examples. Next, we will consider the attention weights in the corpus level. Specifically, we investigate the distribution of the attention weights on all the words in the dataset. Given that the entities and the trigger words are usually near to each other for the relation expression, we only consider the three words around the entities for the weights distribution. In particular, we see the average of the weights as the global attention weight for the word. In Table 8, we give some examples (words that have large attention weight) from the dataset of the tasks. In the table, most of the words can be considered as “trigger words” of the relations. Thus, we can say that the attention mechanism is able to learn some key knowledge of relation expression.

**Table 8:** The top 10 words with large learned attention weight in PPI, DDI, and ChemProt corpora

Task	Word stem
PPI	Activ(ate), complex, associ(ate), interact, human
	Protein, bind, domain, specif(y), receptor
DDI	Concomitantli, combin(e), concomit(ant), increas(e), use
	Concurr(ent), decreas(e), inhibit, receiv(e), administ(er)
ChemProt	Phosphoryl(ate), attenu(ate), stimul(ate), deriv(e), regul(ate)
	Novel, metabol(ize), reduc(e), induc(e), inhibit

For the calculation of global attention weight, we use Porter’s stemmer [32] to obtain the word stem for each word since words might in different forms in the sentence. For example, the stem of “activate” is “activ”, and the words like “activation” and “activates” share the same word stem

## Methods

In this work, we see relation extraction as a classification problem. Specifically, when a sentence and two entity mentions are given, we have to tell if the sentence expresses a specific relation between the two entities. Here we employ the BERT model to solve the relation extraction problem, and we will design approaches to improve the pre-training and fine-tuning of BERT model.

In this section, we first give some background knowledge about BERT model. Then we describe our approach of improving the pre-training of BERT using sub-domain adaptation. Next, we introduce the basics of utilizing edge probing to explore the learned knowledge in different layers of BERT model. We then discuss the SLL fine-tuning of incorporating the knowledge from the last layer. At last, we give the details of our experiment setup and data pre-processing.

### Introduction of BERT

BERT [14] is language representation model using bidirectional transformer [16]. Trained with “masked language model” technique, BERT is able to learn the context of a word from both left and right side in the text (sentence). To apply the BERT model on a specific task, it needs two steps: (1) pre-training on unlabeled text to obtain general knowledge of a domain; (2) fine-tuning on labeled data to gain the specific knowledge of a task.

*Pre-training of BERT* In this stage, BERT can learn a general language representation via two well-designed tasks: masked language model (MLM) and next sentence prediction (NSP). The MLM technique randomly replaces a portion of tokens with a special token ([MASK] token), and lets the language model predict the replaced tokens. In the original BERT, MLM only selects subwords to mask. BioBERT, SciBERT and BlueBERT follow this convention of subwords masking. The PubMedBERT utilizes whole-word masking (WWM) instead, which enforces the mask of the whole word in MLM if one or more of its subwords are chosen. In NSP, the model is trained to predict whether a sentence is followed another sentence in the original text given a sentence pair. The pre-training of BERT usually utilize a great quantity of unlabeled data. In addition, BERT is originally designed for the general domain, and it is pretrained on two datasets: English Wikipedia and BooksCorpus.

BERT for general domain might generalize poorly on a specific domain since every domain has its unique knowledge. BERT can not gain such knowledge without pre-training on the data for specific domain. For instance, the representation of the biomedical entity names from the general BERT model will be of low quality, since the pre-training datasets contains too few such names for the BERT model to generalize well. Thus we need to adapt the BERT model to biomedical domain before applying it to biomedical tasks (a.k.a., pre-training the BERT model on biomedical data). There are two ways of adapting BERT model: (1) continual pre-training using biomedical data from pre-trained BERT for general domain (e.g., BioBERT [17]); (2) pre-training BERT on biomedical data from scratch (e.g., PubMedBERT [20]). Obviously, the difference between these two types of adapted models is whether to use the general domain data before the biomedical domain adaptation. In this paper, we experiment with those two types of adapted BERT models for the biomedical domain.

*Fine-tuning of BERT* The task-agnostic representation (from pre-training) can be fine-tuned for diverse downstream tasks via supervised training on labeled datasets. For a specific task, we just need to attach a custom layer on the last layer of BERT. Next, the BERT parameters can be fine-tuned like supervised learning using the labeled data of a specific task. For our classification tasks, we can build a classifier by appending a softmax layer on the output of [CLS] token. The full name of [CLS] is “classification”, and it is designed for the purpose of classification in BERT. In the input for BERT, the sequence always starts with [CLS] token.

### Improving pre-training using sub-domain data

We can see from Table 2 that the different pre-training data lead to significantly different performance on our focused tasks. Inspired by this observation, we explore whether task-related pre-training data could improve the BERT model. Specifically, we investigate whether adding an extra pre-training step using task-related data after the domain adaptation pre-training to help model generalization. We call it sub-domain adaptation as we are seeking to tailor the model further for specific tasks.

In sub-domain adaptation, we use the following way to extract the sub-domain data that are related to a specific relation extraction task. We assume that the text containing some type(s) of entity in a relation (e.g., protein for the PPI relation) is relevant to the specific relation extraction task. Since PubMed is our main source of pre-training data, we extract the PubMed abstracts containing specific types of entity as the sub-domain data. Specifically, for the PPI task, we extract abstracts from PubMed via the query “Protein OR Gene” and we obtain 7,729,611 abstracts for protein/gene domain using this query. Similarly, the DDI task involves the drug entities, so we use the PubMed query “Drug” and 5,714,799 abstracts are extracted as pre-training data for drug domain. For the ChemProt task, we use Pubtator [33] to extract the PubMed abstracts that contain protein/gene and chemical entities, and 3,375,380 abstracts are used as the sub-domain data.

**Fig. 3:**
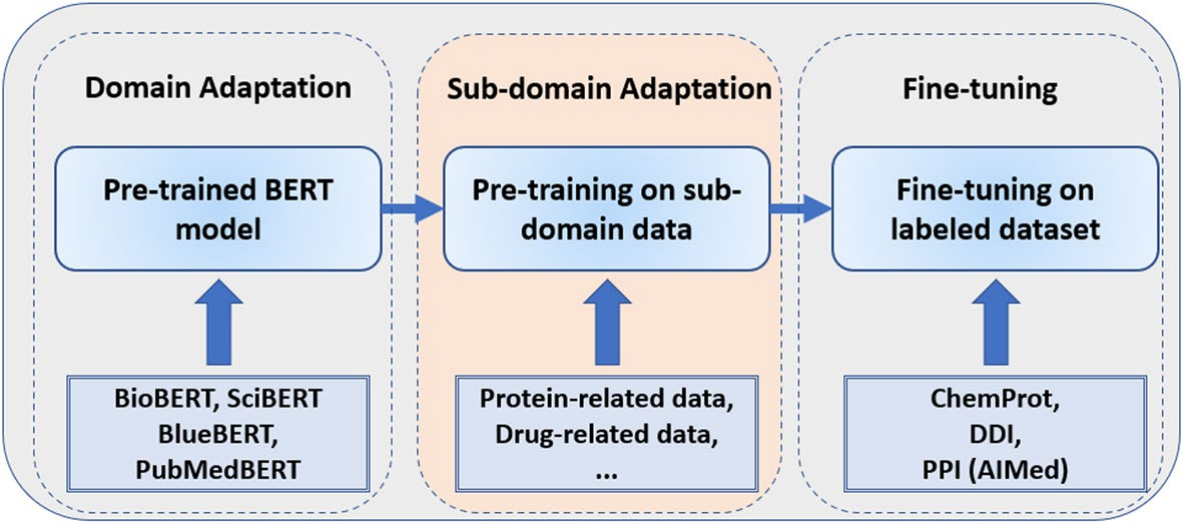
BERT model training process with sub-domain adaptation

The new training process of BERT model is illustrated in Fig. 3. The first box represent the standard creation of BERT models for biomedical domain. The rightmost box also is the standard fine-tuning for the relation extraction tasks. Our experiment here involves the inclusion of sub-domain adaptation in the middle box of Fig. 3.

### Improving the fine-tuning of BERT

As we discussed before, the BERT model for relation extraction classification problem only utilizes the classification ([CLS]) token when it is fine-tuned on the training dataset. If the last layer contains useful information about the task, we should incorporate it to boost the model performance during fine-tuning. In this subsection, we first introduce the edge probing technique and then utilize it to verify the usefulness of the information in the last layer of BERT. Then, we propose two methods to incorporate the information from the last layer in a refined fine-tuning mechanism.

#### Introduction of edge probing

Edge probing is proposed in [28] to measure the quality of encoded information about linguistic structure in a pre-trained encoder (BERT in our case). Specifically, edge probing aims to evaluate how well the word is represented in each position, and what knowledge is learned about the structural information of the sentence. For instance, through edge probing, we can know if the constituent information (like noun phrase or verb phrase) of the words is encoded in the representation from the encoder. To achieve this goal, edge probing converts this problem into classification tasks and builds probing classifiers using the word representations and the expected labels. Based on the performance of the probing classifier, we can measure how well the structural information about that word in the sentence is encoded. Let us take the constituent type as an example, the phrase “is a global brand” in the sentence “The important thing about Disney is that it [is a global brand].” is a verb phrase (VP), so the probing classifier should predict the type “VP” when it is given the representation of “is a global brand”. Usually, we use multi-layer perceptron (MLP) for probing classifier. Also, the parameters of encoder are frozen during the training of probing classifier. For more detailed description of probing classifier on different type of relations, we refer readers to the paper [28].

**Fig. 4:**
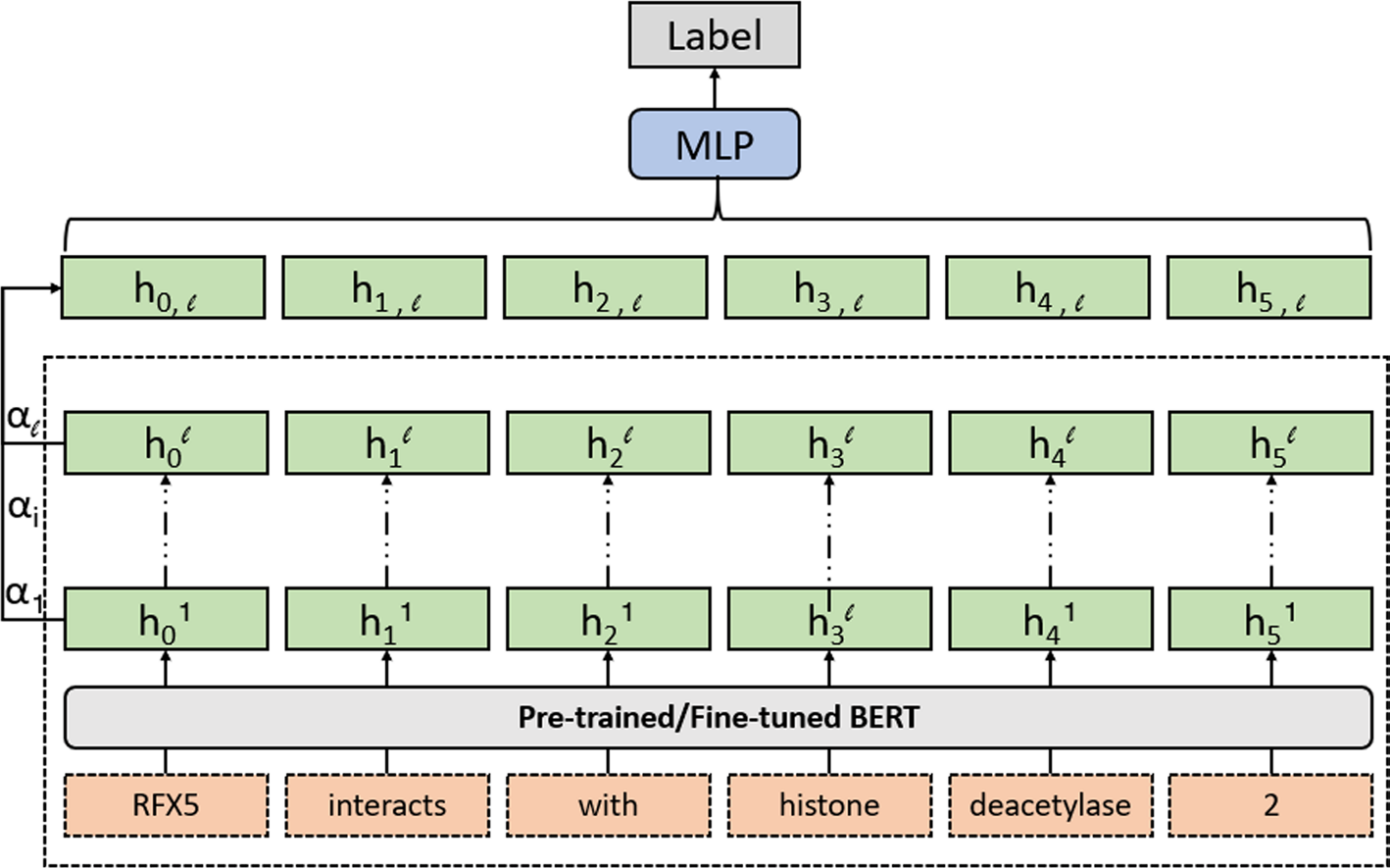
Probing classifier architecture. We freeze the parameters of BERT model during the training of probing classifier. Through the learned $\alpha$, we can know the relevance between each layer and the task. Also, we can tell which layer learns the knowledge for a specific instance by building a series of probing classifier $\{P_\tau ^l\}_{l=1}^L$. For the relation extraction instance “RFX5 interacts with histone deacetylase 2”, if the probing classifier $P_\tau ^l$ predicts the interacting relationship between proteins “RFX5” and “histone deacetylase 2” correctly using the information of the first *l* layers, but $P_\tau ^{l-1}$ does not predict correctly using the information of the first (*l*-1) layers. We can say that the knowledge about this instance is learned in the *l*-th layer

Originally, the edge probing is to investigate the role of words in the sentence in [28]. In [27], the scalar mixing technique [13] is combined with edge probing to explore the encoded information from different layers of the encoder. In particular, through this approach, we can tell which layer(s) are most relevant to a specific task. In formal, let $h^l=[h_0^l,\ h_1^l,\ldots ,\ h_{N}^l]$ be the word representation after *l*-th layer of the encoder, then we can build a probing classifier (Fig. 4) using the first *l* layers:

$$\begin{aligned} h_{i,l}&= \gamma _l \sum ^l_{k=0} \alpha _l^k h_i^k,\ i=1,\ldots ,N \\ P_\tau ^l&= \text {MLP}([h_{0,l},h_{1,l},\ldots ,h_{N,l}]) \end{aligned}$$

where $\alpha _l=softmax([\alpha ^{(0)},\alpha ^{(1)},\ldots ,\alpha ^{(l)}])$ and *N* is the sequence length. During the training, the parameters $\gamma _l$ and $\alpha _l$ will be jointly learned. After the training of $P_\tau ^l$, we can use the learned parameters $\alpha _l$ to estimate the contribution of each layer for our task. If the weight $\alpha ^{(i)}$ for the i-th layer is high, we can say that the i-th layer contains more relevant information about our task. In this work, we use the framework of edge probing to measure the learned knowledge in the layers of BERT model.

#### Investigation of the knowledge in the last layer

In the previous subsection, we demonstrate that the contribution of each layer in BERT can be learned through the weights for layers in a probing classifier. However, those weights are irrelevant to the training data distribution, which means we can not tell how many layers BERT needs to predict a specific instance correctly. This yields a question: what knowledge about the training data is only learned in the *l*-th layer, but not learned in the first (*l*-1) layer(s)?

Similar to the exploration in [27], the above problem can be addressed by training a series of probing classifier $\{P_\tau ^l\}_{l=1}^L$, where *L* is the total layers of the encoder. The classifier $P_\tau ^l$ utilizes all the information from the first *l* layers, while the knowledge in the first *l*-1 layers is employed in the classifier $P_\tau ^{l-1}$. Therefore, the difference of performance score (F1 score) $\Delta _\tau ^l$ on test data between $P_\tau ^l$ and $P_\tau ^{l-1}$ stands for the learned knowledge only from the *l*-th layer:

$$\begin{aligned} \Delta _\tau ^l=Score(P_\tau ^l)-Score(P_\tau ^{l-1}) \end{aligned}$$

After calculating $\{\Delta _\tau ^l\}_{l=1}^L$, we can acquire the distribution of the learned knowledge from the training data in each layer.

Our purpose is to investigate whether the last layer contains useful information for our tasks, so here we build the probing classifiers using the fine-tuned BioBERT model [17]. During training, we freeze the weights of BioBERT and only adjust the parameters of the probing classifiers. We show the score $\Delta _\tau ^{L}$ (measurement of knowledge) of the last layer of BioBERT for the datasets of our three tasks in Fig. 1. We can see that the last layer (12-th layer for the BERT_base_ model) contains useful information in all cases and it is necessary to incorporate the discarded knowledge during fine-tuning.

#### Refined fine-tuning for the BERT model

We have shown that the fine-tuning method of only using classification token (CLS) discards some useful information in the last layer. Also, during the pre-training of BERT, the [CLS] token is only utilized in the next sentence prediction (NSP) task. This means that the [CLS] token might not encode the information about the interaction between entities because it is not designed to gain this type of information. In this subsection, we describe the mechanism of incorporating all the available information of the last layer in the BERT fine-tuning process. The proposed mechanism is called SLL fine-tuning: fine-tuning with information summarization in the last layer of BERT.

**Fig. 5:**
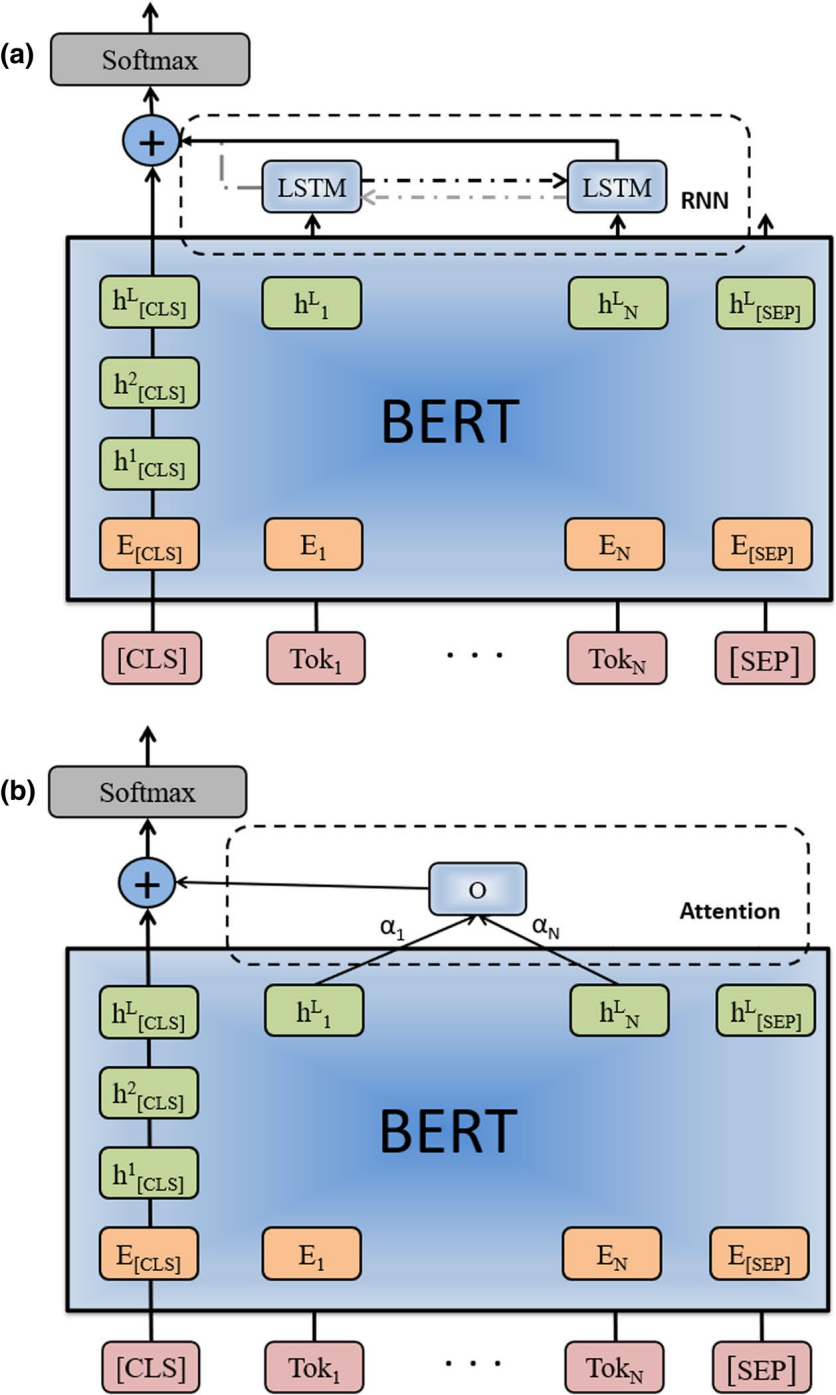
Model architectures after incorporating all outputs from the last layer. In **a** we show both LSTM (only black in the RNN box) and biLSTM (both black and grey line in the RNN box). **a** RNN on the last layer. **b** Attention on the last layer

Our method includes two steps: (1) summarizing the ignored outputs in the last layer; (2) concatenating the summarized knowledge to the [CLS] output as final output from BERT. In step 1, we explore two types of methods of summarizing the information of the last layer: recurrent neural network (LSTM [34] and biLSTM [35]) and attention mechanism. In Fig. 5, we show the BERT model architectures after appending these two methods on the outputs of the last layer.

Formally, let H be the dimension of hidden states and L be the layer number of BERT model, then all the information in the last layer can be represented:

$$\begin{aligned} h_{all}^L=\big \{h_{CLS}^L,\ h_1^L,\ h_2^L,\ \ldots ,\ h_{N}^L,\ h_{SEP}^L\big \} \end{aligned}$$

where $h_{CLS}^L$ and $h_{SEP}^L$ are the classification token output and separation token output, respectively. Previously, only $h_{CLS}^L$ is used for classification problem during fine-tuning. Here we first summarize the discarded information in the last layer, i.e., $h^L=\{h_1^L,\ h_2^L,\ \ldots ,\ h_{N}^L\}$, (the sentences separation token $h_{SEP}^L$ is ignored here) using the RNN sequence model and attention mechanism:

1. The first choice of model to summarize a sequence is the recurrent neural network. Among RNN models, we choose LSTM because it handles the “long-term dependencies” better. Also, we utilize biLSTM to summarize the sequence in both forward and backward directions. We take the output of last LSTM unit as the representation of our sequence in LSTM. As for biLSTM, we just concatenate the outputs of first unit in backward direction and the last unit in forward direction as the final representation:
  $$\begin{aligned} O=\Bigg \{ \begin{array}{l} LSTM(h_i^L) \qquad \qquad \qquad \qquad (LSTM) \\ LSTM(\overrightarrow{h_i}^L) \oplus LSTM(\overleftarrow{h_i}^L) \quad (biLSTM) \end{array} \end{aligned}$$
2. Attention mechanism is another option of summarizing sequence data, which assigns a weight to each component. In particular, we employ the additive attention to summarize the sequence:
  $$\begin{aligned}[\alpha _i] = softmax(h^LK) \end{aligned}$$
  $$\begin{aligned} O = \sum ^N_{i=1}\alpha _i h_i^L \end{aligned}$$
  where $K^{H \times 1}$ are trainable parameters.

After the summarization, the output from RNN/attention mechanism is concatenated to the [CLS] output, and this combined output is the final output of BERT:

$$\begin{aligned} h =h_{CLS}^L \oplus O. \end{aligned}$$

Then, we put a softmax layer on top of this representation to calculate the probability distribution on the labels:

$$\begin{aligned} p=softmax(W_fh+b_f) \end{aligned}$$

where $W_f^{C \times H}$, $b_f^{C \times 1}$ are trainable parameters. *C* denotes the number of categories (classes) of our tasks.

In all the fine-tuning mechanisms we described so far, they all involve the use of [CLS] token. It is necessary to investigate the role of [CLS] token and measure its contribution on the classification tasks. Thus we experiment with the fine-tuning process in which [CLS] token is removed from the final output. In formal, we only use $h =[O]$ as the final output of the BERT model for the input. Then, the same softmax layer $p=softmax(W_fh+b_f)$ is utilized for predicting the relation type. The experiments using these two different fine-tuning methods can help interpret the roles of the [CLS] output and sentence outputs when the BERT model is applied on classification task.

### Combining the techniques for pre-training and fine-tuning

We have proposed techniques to improve the BERT model in both the pre-training and fine-tuning stage, so a natural idea is to combine these two techniques. For the new BERT model, we add an extra step for sub-domain adaptation in the pre-training and utilize the SLL mechanism in fine-tuning stage. In this way, we take the full advantage of the knowledge in the sub-domain data and the learned information in the last layer of BERT model.

### Experiment setup

For the sub-domain adaptation, we train the BERT with 100K steps on the sub-domain data using maximum sequence length of 128, learning rate of 2e-5, and batch size of 192 in our experiments. In the sub-domain pre-training for BioBERT, we follow its settings in masked language model task and only utilize subwords masking. In contrast, we employ whole word masking in the pre-training of PubMedBERT. We employ Google Cloud TPU (v3-8) for the sub-domain pre-training, and it takes about 4 h for the pre-training of each sub-domain.

For the BioBERT model fine-tuning, we use maximum sequence length of 128, training epoch of 10, learning rate of 2e–5, and batch size of 32. For the fine-tuning of PubMedBERT model, maximum sequence length of 256, training epoch of 10, learning rate of 2e–5, and batch size of 16 are utilized. In the SLL fine-tuning, we use the hidden size of 768 in the LSTM and biLSTM model, and the sequence length is 128 on BioBERT outputs and 256 on PubMedBERT outputs. In the edge probing experiments, we use a two-layer perceptron as the probing classifier, and 1024 as the hidden layer unit number. During training of probing classifiers, we use learning rate of 2e–5 and training epoch of 4. For the fine-tuning of BERT and the training of probing classifiers, we train the models on GeForce RTX 2080Ti GPU. The fine-tuning takes about 10 h, 2 h, and 3 h on the PPI, DDI, and ChemProt tasks respectively.

We implement our experiments using Tensorflow [36]. Our data and code are publicly available at: https://github.com/udel-biotm-lab/BERT-RE.

### Data pre-processing

As illustrated before, one relation extraction instance includes two components: the text (sentence) and the entities in it. With the aim of making BERT model recognize the position of the biomedical entities, we follow the standard pre-processing step for relation extraction to replace the entity names with some predefined tags. For example, the protein names are replaced with the tag “@PROTEIN$”. Similarly, we replace the drug names and the chemical names with “@DRUG$” and “@CHEMICAL$”, respectively. In Table 9, we give some pre-processed instances from the corpora.

**Table 9:** Pre-processed examples for the three tasks

Task	Label	Sentence examples
PPI	Positive	Nuclear protein @PROTEIN$ is a coactivator for
		the transcription factor @PROTEIN$.
	Negative	Their order of selection was @PROTEIN$ effusion,
		@PROTEIN$ serum, TNFalpha-effusion, and C3 effusion.
DDI	EFFECT	@DRUG$ may increase the ototoxic potential of other drugs
		such as aminoglycoside and some @DRUG$.
	MECHANISM	Cimetidine: @DRUG$ increases @DRUG$ plasma levels.
ChemProt	CPR:6	We conclude that @CHEMICAL$ and BAAM are competitive
		slowly reversible @PROTEIN$ antagonists on rat left atria.
	CPR:9	@PROTEIN$ plays a role in purine salvage by catalyzing the
		direct conversion of adenine to @CHEMICAL$.

## Conclusion

In this paper, we proposed two techniques to improve the pre-training and fine-tuning of BERT model. We first utilized the sub-domain data in the pre-training phase of BERT model to help its generalization on different tasks. Then we proposed a refined fine-tune process to utilize all the knowledge in the last layer of BERT model. In addition, we explored the combination of those two techniques. We demonstrated that the proposed methods are effective on three widely studied relation extraction tasks. Furthermore, the experiment results showed that the proposed methods achieve better performance on all three tasks and we achieved state-of-the-art performance on three relation extraction benchmark sets. In the future, we will apply our method on other relation extraction tasks in biomedical domain.

## Data Availability

The data and code are publicly available at: https://github.com/udel-biotm-lab/BERT-RE.

## Abbreviations

BERT: Bidirectional encoder representations from transformers
RE: Relation extraction
PPI: Protein–protein interactions
DDI: Drug–drug interactions
ChemProt: Chemical–protein interactions
P/G: Protein or gene
D: Drug
CP: Chemical and protein
NLP: Natural language processing
RNN: Recurrent neural network
LSTM: Long short-term memory
BiLSTM: Bi-directional long short-term memory
SLL: Summarization in the last layer
MLM: Masked language model
NSP: Next sentence prediction
CLS: Classification
Att: Attention mechanism
LL: Last layer
P: Precision
R: Recall
F: F1 score
